# Intimate Injection Partnerships Are at Elevated Risk of High-Risk Injecting: A Multi-Level Longitudinal Study of HCV-Serodiscordant Injection Partnerships in San Francisco, CA

**DOI:** 10.1371/journal.pone.0109282

**Published:** 2014-10-06

**Authors:** Meghan D. Morris, Jennifer Evans, Martha Montgomery, Michelle Yu, Alya Briceno, Kimberly Page, Judith A. Hahn

**Affiliations:** 1 Department of Epidemiology and Biostatistics, University of California San Francisco, San Francisco, CA, United States of America; 2 Joint Medical Program and Program in Medical Education for the Urban Underserved, University of California San Francisco & University of California, Berkeley, San Francisco, CA, United States of America; 3 University of New Mexico Health Sciences Center, Albuquerque, New Mexico, United States of America; 4 Department of Medicine, University of California San Francisco, San Francisco, CA, United States of America; Rollins School of Public Health, Emory University, United States of America

## Abstract

**Background:**

It is increasingly recognized that the risk for HIV and hepatitis C (HCV) transmission among people who inject drugs (PWID), such as syringe sharing, occurs in the context of relationships between (at least) two people. Evidence suggests that the risk associated with injection behavior varies with injection partner types.

**Methods:**

We utilized longitudinal dyad-level data from a study of young PWID from San Francisco (2006 to 2013) to investigate the relationship-level factors influencing high-risk injecting within HCV-serodiscordant injection partners (i.e., individuals who injected together ≥5 times in the prior month). Utilizing data from 70 HCV-serodiscordant injection partnerships, we used generalized linear models to examine relationship-level predictors (i.e., partnership composition, partnership closeness, and partnership dynamics) of: (1) receptive syringe sharing (RSS); and (2) receptive cooker use (RCU), as reported by the HCV-negative injection partner.

**Results:**

As reported by the “at-risk” HCV-negative injection partner, receptive syringe sharing (RSS) and receptive cooker use (RCU) were 19% and 33% at enrollment, and 11% and 12% over all visits (total follow-up time 55 person-years) resulting in 13 new HCV-infections (incidence rate: 23.8/100 person-years). Person-level factors, injection partnership composition, and partnership dynamics were not significantly associated with either RSS or RCU. Instead, intimate injection partnerships (those who lived together and were also in a sexual relationship) were independently associated with a 5-times greater risk of both RSS and a 7-times greater risk of RCU when compared to injecting only partnerships.

**Conclusion:**

Our findings suggest a positive, and amplified effect of relationship factors on injecting drug risk behaviors among young PWID injection partnerships. The majority of interventions to reduce injection drug use related harms focus on individual-based education to increase drug use knowledge. Our findings support the need to expand harm reduction strategies to relationship-based messaging and interventions.

## Introduction

Hepatitis C virus (HCV) is more efficiently transmitted through intravenous drug use than HIV [1]–[3] and can be transmitted through sharing of injection equipment (e.g., cookers (a container used to mix drug into an injectable liquid), cottons, and rinse water) [4], [5]. The identification of key environmental, social, and individual level factors associated with syringe sharing has resulted in the development of both behavioral and structural interventions to reduce transmission of HIV and HCV in people who inject drugs (PWID) [6]–[8]. Even with the wide-spread application of knowledge-based interventions, peer-education models, and syringe exchange programs high-risk injecting persists with 50–70% of PWID reporting sharing [9]–[12]. These injection behaviors put PWID at risk for HIV and resulting in PWID having the highest burden of hepatitis C virus (HCV) in the U.S. and globally [13], [14]. Interventions that also target relationship-level factors may further reduce intravenous transmission of HCV; as has been shown by HIV prevention researchers to reduce sexual transmission of HIV [15], [16]. For example, by applying understanding about relationship dynamics couples counseling has been shown to increase HIV testing and condom use [15], [17]–[19].

Previous studies have consistently shown that female PWID are at greater risk of syringe and injection equipment sharing, referred to hereafter as high-risk injecting behaviors, than their male counterparts [20]–[22]. Furthermore, females are more likely to be initiated into injection drug use by a male sex partner [23], frequently reliant on male injection partners for both drugs and injection equipment [24], and are often second to use the syringe [20], [25] suggesting the underlying influence of interpersonal factors. A few previous studies found the odds of high-risk behaviors are greatest between individuals with close social ties, such as those in sexual partnerships and family members, or between individuals who were dissimilar in both gender and age [6], [26]–[28]. However, these studies rely on single-level cross-sectional data with few relationship-level measures.

We conducted a novel longitudinal study enrolling HCV-serodiscordant injection partners to examine HCV transmission and dyad-level risks. We examined the association of a series of relationship measures including partnership gender and age composition, relationship closeness, and relationship dynamics with two high-risk injecting behaviors: (1) receptive syringe sharing (RSS) and (2) receptive cooker sharing (RCU). HCV-serodiscordant injection partnerships are focal points for HCV transmission, thereby allowing a targeted study of key determinants of behaviors placing individuals at risk for infection.

## Methods

### Ethics Statement

We included minors between the ages of 15–18 years in our study. Minors represent a special population, and our outreach team has long experience with young PWID. In prior years, approximately three percent of our study population has been aged 15–18. All participants under age 18 in our study population are defined as emancipated minors by service provision agencies. None resided with their parents, and all are financially independent. Due to the illicit nature of the primary behavior at the center of this study, informing parents is not a reasonable requirement to protect these potential subjects and may in fact cause subjects harm. During data collection data were anonymized by assigning a random unique identifier to each participant. To protect the identity of participants The University of California, San Francisco Ethics Board approved the consent procedures and the study protocols. All participants gave written informed consent prior to their inclusion in the study.

### Study Design

**Figure 1:**
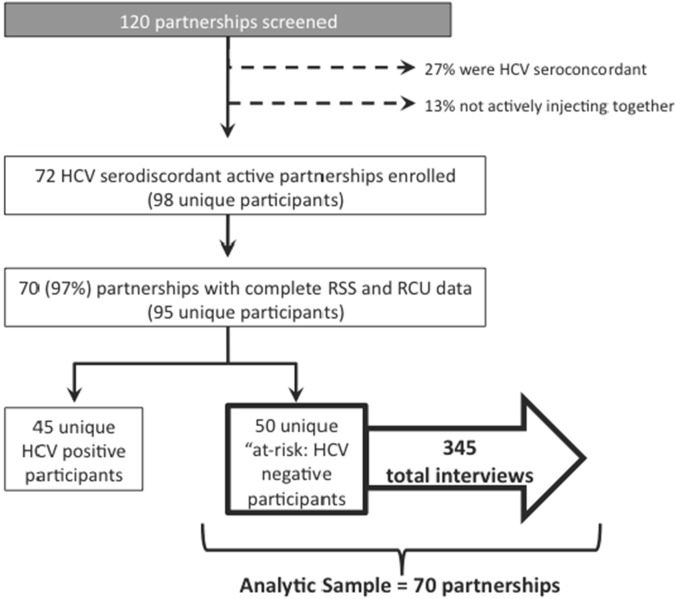
Flow chart of total study population and analytic sample.

From January 2006 to July 2013, study participants were recruited from the UFO Study in San Francisco, CA, USA a prospective study of HCV transmission in young (<30 years) PWID [9], [29]. UFO study participants were invited to recruit individuals with whom they were currently injecting drugs to participate in a prospective sub-study of HCV transmission within HCV-serodiscordant injection partnerships. Injection partnerships were eligible for the sub-study enrollment if (1) individuals injected together within the same physical space at least 5 times in the prior month, and (2) partners were HCV infection discordant (i.e., one injection partner was HCV-positive (referred to as “HCV positive” partner herein) and the other was HCV-negative (referred to as “at-risk” partner herein) (figure 1). Eligibility did not require that drugs or injecting equipment were shared. Participants of the UFO Study who reported injecting at least weekly with a person of HCV discordant or unknown serostatus were invited to return with up to three of their injection partners within 4 weeks to screen for the Partner Study eligibility. Additional information on UFO Partner Study recruitment has been published previously [30].

Injection partnerships were verified through a series of screening questions, rotated regularly, administered separately prior to study enrollment, asking each injection partner to provide basic demographic and injecting behavioral information about themselves and their injection partner. This technique was adapted from a study that recruited drug using couples [31]. Answers were crosschecked and a minimum of 5/7 answers was required for dyad eligibility. Eligible and consenting participants individually completed an interviewer-administered survey at baseline and at monthly follow-ups for a minimum of 6 months or until injection partnerships ended, whichever came later. The prospective nature of data collection resulted in information on current partnerships only. Retention rates for the 1-, and 3-month visits were 86%, and 78% respectively. All protocols were reviewed and approved by the Institutional Review Board of the University of California, San Francisco.

### Measures

#### Person-level measures

Person-level demographic characteristics included gender, age at enrollment, self-identified race, education level, recent homelessness or unstable housing (e.g., slept on street, park, shelter, street), lifetime history of jail/prison, and lifetime participation in drug treatment. Information was collected on person-level recent (prior 30 days) drug use information (injection and non-injection drug use), and other key drug use characteristics (e.g., age first injected, drug injected most often).

Hepatitis C virus serostatus was determined using serum samples from baseline and quarterly visits. Samples were tested for anti-HCV using HCV enzyme immunoassay EIA 3.0 (Ortho ELISA 3, Hercules, CA) or HCV rapid test (OraSure Technologies: Bethlehem, PA). Reactive samples were tested for HCV RNA using a nucleic acid amplification test, the discriminatory HCV transcription-mediated amplification assay component of the Procleix human immunodeficiency virus type 1 (HIV-1)/HCV assay (Gen-Probe Inc, San Diego, CA; Novartis Vaccines and Diagnostics, Emeryville, CA). HCV test results were disclosed separately to each member of the injection partnership. Participants who were positive for anti-HCV or HCV RNA were considered HCV positive.

#### Injection partnership-level measures

Previous studies [32]–[34] have shown that females and younger PWID are at higher odds of engaging in syringe and ancillary equipment sharing, thus we were interested in looking at the influence of age and gender composition of the partnerships on the risk of RSS and RCU. Two partnership composition variables comprised of age and gender differences as well as which partner was “at risk” versus HCV positive, were constructed: (1) Gender mix/HCV status was represented by a four-level nominal variable for HCV status and gender of each partner within injection partnerships, defined as follows: HCV− male/HCV+ male; HCV− male/HCV+ female, and HCV− female/HCV+ male, HCV− female/HCV+ female; and (2) similarly, age difference/HCV status was defined as follows: HCV− partner ≥3 years older than HCV+ partner, HCV− partner +/−3 years of HCV+ partner, HCV− partner ≥3 years younger. We hypothesized that female-male partnerships and partnerships with larger age gaps across partners would be associated with higher RSS and RCU.

For most PWID, injecting drugs is a highly social behavior (47); to examine the influence of interpersonal factors we examined six items as measures of relationship closeness/depth: (1) engaged in vaginal or anal sex with the injection partner in the prior month; (2) a three-level ordinal measure for prior month cohabitation (did not cohabitate, cohabitated 1–28 days, and cohabitated >28 days) was created based on the following item responses: “did you stay together in the same tent, squat, shelter, apartment, whatever for at least one night” and if so, “how many nights did you stay together?”; (3) duration (months) of time knew injection partner; (4) duration (months) previously injected with injection partner; (5) injected only with the injection partner in the prior month; and (6) number of study visits the injection partnership completed together. Additionally, several measures of partnership-level injection behaviors were examined.

#### Dependent Variables

The two injection partnership-level binary outcomes of interest were: (1) recent RSS, measured by “within the prior 30 days, did you inject with a syringe/needle that your injection partner had already used, even if by accident or mistake?” and (2) recent RCU, measured by “within the prior 30 days, was there any time that your injection partner’s previously used syringe/needle had been used with a cooker (or other container used to dissolve drugs) before you used that cooker?”.

### Analyses

The HCV uninfected injection partner is referred to as the “at-risk” injection partner and the HCV infected injection partner is referred to as the HCV-positive injection partner. We calculated the baseline prevalence of recent receptive syringe sharing (RSS) and recent receptive cooker use (RCU) among the “at-risk” HCV-negative injection partners. To model directional risk of HCV transmission, we utilized data from only the “at-risk” HCV-negative partner collected at multiple time-points prospectively. Models accounting for three-levels of clustering failed to converge, likely due to small sample size. Our final modeling approach accounts for dependence due to partnerships measured at multiple time points (two levels of clustering) rather than the dependence due to individuals in multiple partnerships, as we did not find the latter to have a strong influence on variance estimates. We used generalized estimating equations (GEE) model to model the outcomes using the exchangeable correlation structure, the logit link function, and robust standard errors [35]. This approach allowed each HCV-discordant injection partnership to represent individual partnerships.

We constructed such models to examine single variable associations of the person- and partnership-level measures with the two outcome variables. Overall, data was complete for 97% of our sample across all time points. We then included recent cohabitation and sexual relationship in a multivariable model. However, we found that the recent cohabitation and sex within injection partnerships variables were statistically significant individually but not when together highly collinear when entered simultaneously into a multivariable model. Therefore we constructed a composite variable defined as: injection partnerships that did not cohabitate, injection partnerships who lived together 1–28 days, injection partnerships that cohabitated ≥28 days, sexual injection partnerships that cohabitated 1–28 days, and sexual injection partnerships that cohabitated ≥28 days. There were no sexual injection partnerships that did not cohabitate for at least one night; therefore this variable had 5 levels. Final multivariable models were constructed by first entering this composite variable. Additional variables found to be significant at the P≤0.20 level in bivariate analyses were then entered using a manual forward step-wise method. We also included the number of interviews completed to adjust for the influence of study participation on self-reported risk behaviors previously observed [36].

## Results

### Sample description

A total of 95 individuals of 98 (96%) enrolled study participants had complete data on both high-risk injecting behaviors, and reported being in an active injection partnership with their injection partner (figure 1). The 70 HCV-discordant injection partnerships were comprised of 95 unique persons; of whom 50 were HCV-negative at baseline. About two-thirds (65%) were members of 1 partnership, 20% were members of 2 partnerships, 10% were members of 3 partnerships, and 5% were a member of either 4, 5, or 6 partnerships. However, each member of the partnership contributed data for each partnership at each time point. Directional risk was assessed prospectively based on a total of 345 interviews from the “at-risk” HCV-negative injection partner interviews. High-risk injecting behaviors within the prior 30 days were as follows: 19% of HCV-negative “at-risk” injection partners reported receptive syringe sharing (RSS), and 33% reported receptive cooker use (RCU) at enrollment; and 11% and 12% reported RSS and RCU, respectively, across all study visits. We observed 13 new HCV infections among the HCV-negative injection partners over a total of 55 person years, for an infection rate of 23.8/100 person-years (95% Confidence Interval [95% CI], 13.8–40.9). Table 1 provides a description of baseline person-level characteristics of the 50 unique “at-risk” HCV-negative injection partners.

**Table 1:** Characteristics of the HCV-negative “at-risk” injection partner at enrollment (n = 50)‡.

	n (%) *or* median (IQR)
**Demographic Characteristics**	
**Gender**	
Male	23 (46)
Female	27 (54)
**Age (years)**	24 (22–27)
**Age**	
16 to 24 years	41 (59)
25 to 30 years	27 (38)
31 to 44 years	2 (3)
**Race**	
White	36 (72)
Non-white/mixed race/other	14 (28)
**Completed High School**	
No	14 (28)
Yes	36 (72)
**Homeless/marginally housed, prior 30 days**	
No	22 (53)
Yes	20 (47)
**Ever incarcerated (jail or prison)**	
No	7 (14)
Yes	43 (86)
**Ever in drug treatment**	
No	10 (20)
Yes	30 (80)
**Self-reported HIV status (n = 73)**	
Negative	36 (92)
Positive	1 (3)
Indeterminate	2 (5)
**Drug and alcohol use**	
**Drank alcohol daily or almost daily** †	
No	39 (78)
Yes	11 (22)
**Smoked crack cocaine** †	
No	11 (22)
Yes	39 (78)
**Snorted or smoked cocaine** †	
No	22 (44)
Yes	22 (44)
**Snorted or smoked methamphetamine** †	
No	13 (26)
Yes	37 (74)
**Age (in years) first injected drugs years**	18 (16–20)
**Age first injected drugs**	
10 to 14 years old	4 (8)
15 to 18 years old	24 (48)
19 to 38 years old††	22 (44)
**Number of days injected, prior month**	25 (7–30)
**Number of people with whom injected other than partner, prior month**	3 (1–5)
**Drug injected most often, prior month**	
Heroin	29 (63)
Stimulant	18 (27)
Other	3 (6)

‡ Data represent unique injection partners as individuals could enroll with multiple injection partners.

† Prior 3-months.

†† Age <30 when enrolled in parent study; a small number of participants were age >30 at enrollment in partner study.

At enrollment, the majority of injection partnerships were composed of an HCV+ male and HCV− female (53%) (Table 2), with 9% of injection partnerships composed of an HCV+ female and HCV− male, 31% HCV-serodiscordant male-male, and only 7% HCV-serodiscordant female-female. Half (50%) of the injection partnerships comprised of the “at-risk” injection partner being ≥3 years younger. Approximately one-third (36%) engaged in sexual behaviors within the prior month; most (79%) cohabited at least one night together within the prior month; the median number of months injection partners had known each other was 10 (Interquartile Range [IQR]: 4, 24); and the median number of months injecting together was 4.5 (IQR: 1.5–12). Only 11% reported injecting exclusively with the enrolled partner; among whom the majority were in cohabitating sexual injection partnerships (data not shown). Key partnership-level injecting behaviors were as follows: the median number of days injected together within the prior month was 8 (IQR: 3, 22), 29% were recently injected by their “HCV-positive” injection partner, 36% reported their injection partner prepares the drugs most often, and half always pooled money with their injection partner to purchase drugs.

**Table 2:** Injection partnership-level characteristics and behaviors at enrollment, reported by the at-risk HCV-negative partner (n = 70).

Injection partnership-Level Characteristic	n (%) or median (IQR)
**Partnership Characteristics**	
**Gender/HCV composition**	
Male-male injection partnerships	22 (31)
Male HCV+, female HCV−	37 (53)
Female HCV+, male HCV−	6 (9)
Female-female injection partnerships	5 (7)
**Age/HCV composition**	
HCV− injection partner ≥3 years younger	35 (50)
HCV− +/−3 years of HCV+ injection partner	25 (36)
HCV− injection partner ≥3 years older	10 (14)
**Injection partnership also engaged in sexual behaviors** †	
No	44 (64)
Yes	25 (36)
**Type of sexual relationship (n = 25)**	
Main sex partner	23
Casual or occasional sex partner	2
Someone who paid for sex	0
Someone who exchanged drugs for sex	0
**Cohabitation with injection partner (at least one stay together)** †	
No	15 (21)
Yes	55 (79)
**Cohabitation with injection partner (days)** †	
Did not cohabitate in prior month	15 (21)
Cohabitated 1–28 days in prior month	43 (61)
Cohabitated ≥28 days in prior month	12 (17)
**Duration knew injection partner (months)**	10 (4–24)
**Knew injection partner <1 year**	
No	22 (31)
Yes	48 (69)
**Duration injecting with partner (months)**	4.5 (1.5–12)
Injected together for ≤1 month	15 (21)
Injected together for 1 to 6 months	25 (36)
Injected together for 6 to 12 months	17 (24)
Injected together for more than 12 months	13 (19)
**Number of injection partners (excluding enrolled partner)** †	
Partner is only person participant reports injecting with	8 (11)
Participant injected with 1–3 other people	29 (41)
Participant injected with 4 or more other people	33 (47)
**Number of study visits injection partnership completed**	3 (2–4)
**Partnership Injecting Behaviors**	
**Number of days injected with partner** †	8 (3–22)
**Frequency of injecting with partner** †	
Less than once a day	17 (25)
More than once a day	52 (75)
**Injected partner in past month**	
No	56 (80)
Yes	14 (20)
**Injected by partner in past month**	
No	50 (71)
Yes	20 (29)
**Injection partner prepares drugs most often** †	
No	45 (64)
Yes	25 (36)
**Always pool money with injection partner to purchase drugs** †	
** No**	35 (50)
** Yes**	35 (50)
**Injected injection partner’s drug residue** †	
No	45 (65)
Yes	25 (35)
**Participant backloaded his/her syringe with injection partner’s** †	
No	28 (41)
Yes	41 (59)
**Used injection partner’s syringe (RSS)** †	
No	57 (81)
Yes	13 (19)
**Shared injection partner’s cooker** **(RCU)** †	
No	47 (67)
Yes	23 (33)

† within the prior 30 days.

### Bivariate associations

Table 3 presents unadjusted associations between individual- and partnership-level characteristics and recent RSS and recent RCU with “HCV-positive” injection partners. None of the person-level variables or injection partnership composition characteristics were associated with either risk outcome. Instead, several relationship characteristics were associated with both RSS and RCU. Recent sexual intercourse with one’s injection partner was significantly associated with a 3-times greater risk of recent RSS (95% CI 1.43–6.04) and 2.5-fold increased risk of RCU (95% CI: 1.31–4.80). Injection partnerships who cohabitated more days together in the past month were at increased risk of both RSS and RCU compared to injection partnerships who did not recently cohabitate. Lastly, injection partnerships where the “at-risk” HCV- partner recently injected his/her partner’s drug residue was at a 4-fold increased risk of RSS and RCU (95% CI: 1.76–10.08; and 1.84–9.45 respectively) and those who backloaded drugs into his/her syringe using partner’s previously used syringe were a 2-fold increased risk of RSS and RCU (95% CI: 1.17–4.38 and 1.31–4.27 respectively) compared to “at-risk” HCV-negative partners who did not engage in these partnership injecting behaviors.

**Table 3:** Bivariate associations for characteristics associated with recent receptive syringe and cooker use.

	Recent Receptive Syringe Sharing(n = 345)		Recent Receptive Cooker Use(n = 345)	
	RR	95% CI	RR	95% CI
**Person-level characteristics of the “at-risk” HCV-negative injection partner**				
Female vs. male gender	1.30	0.50–3.40	1.50	0.66–3.41
Age at enrollment				
16 to 24 years	ref		ref	
25 to 30 years	0.94	0.40–2.21	0.80	0.30–1.76
31 to 44 years	2.70	0.49–15.12	2.20	0.41–11.68
Non-white vs. white	1.80	0.70–4.80	1.90	0.83–4.24
Completed High School	1.00	0.40–2.72	1.00	0.44–2.48
Homeless/marginally housed†	1.18	0.60–2.39	1.62	0.81–3.26
Ever been in jail/prison	1.45	0.44–4.76	1.50	0.50–4.90
Ever in drug treatment	0.59	0.23–1.54	0.69	0.30–1.60
Age initiated injection drug use				
10 to 14 years old	ref		ref	
15 to 18 years old	0.32	0.08–1.30	0.29	009–0.95
19 to 38 years old	0.50	0.11–2.00	0.66	0.21–2.06
**Partnership characteristics**				
Gender/HCV composition				
HCV-serodiscordant male-male	ref		ref	
Male HCV+, female HCV−	1.51	0.41–5.58	0.90	0.30–2.08
Female HCV+, male HCV−	3.02	0.55–14.03	1.80	0.49–6.12
Age/HCV composition				
HCV- injection partner ≥3 years younger	0.81	0.10–5.40	4.10	0.65–28.55
HCV- injection partner +/−3 years of injection partner	ref		ref	
HCV- injection partner ≥3 years older	0.93	0.19–4.80	3.52	0.81–15.63
Sex with injection partner†	2.94	1.43–6.04**	2.50	1.31–4.80**
Cohabitated with injection partner†				
No cohabitation in prior month	ref		ref	
Cohabited 1–28 days in prior month	2.85	1.38–5.91**	4.61	2.10–10.36***
Cohabited ≥28 days in prior month	5.30	1.90–14.72**	5.35	1.85–15.40**
Known injection partner <1 year				
Duration injecting with partner				
< = 1 month	0.94	0.35–2.5	2.66	0.81–8.71
1–6 month	1.21	0.38–3.83	2.73	0.74–10.05
7–12 month	0.40	0.07–2.13	0.97	0.19–5.06
>12 months	ref		ref	
Injecting Network				
Partner is only person participant reports injecting with†	ref		ref	
Participant injected with 1–3 other people†	0.98	0.52–1.88	1.21	0.54–2.72
Participant injected with 4 or more other people†	0.59	0.25–1.43	0.95	0.35–2.56
Number of study visits partnership completed(per study visit)	0.93	0.86–1.00*	0.91	0.82–1.00*
**Partnership injecting behaviors**				
Injected with partner more than 1x/day (vs. 1x/day)†	1.23	0.64–2.42	1.33	0.45–2.10
Injected by partner most often†	1.63	0.70–3.80	1.85	0.86–3.98
Injection partner prepares drugs most often†	0.86	0.36–2.06	0.77	0.35–1.65
Always pooled money to pay for drugs†	1.58	0.84–2.98	1.66	0.89–3.18
Participant injected partner’s drug residue	4.21	1.76–10.08***	4.20	1.84–9.45***
Participant backloaded drugs into his/her syringeusing partner’s previously used syringe	2.27	1.17–4.38**	2.37	1.31–4.27**

Note: Data from 345 HCV-negative observations representing 70 unique partnerships followed monthly for 6-months or until inactive.

† prior 30 days.

*p≤0.10.

**p≤0.05.

***<0.001.

### Multivariable models

Compared to injection-only HCV-serodiscordant partnerships, the relative risk of the “at-risk” HCV negative injection partner engaging in both recent receptive syringe sharing (RSS) and recent receptive cooker use (RCU) was significantly greater for injection partnerships who were also in sexual relationships cohabitated together (table 4). The relative risk of RSS increased for injection partnerships with each additional relationship layer: Injection partnerships who also cohabitated daily in the past month were at almost 5-fold greater risk of RSS [adjusted risk ratio [ARR] 4.90, 95% CI: 1.01–24.30)] whereas injection partnerships who cohabitated daily in the past month and in a sexual relationship were at 5.5-fold increase of RSS [ARR: 5.45, 95% CI: 1.72–17.18]. Injection partnerships who also cohabitated daily were at 8.5-fold greater risk of RCU and cohabitating injection partnerships who were also engaged in a sexual relationship were at a 7.4-fold increased risk of RCU [95% CI: 1.55–46.35 and 1.95–28.04 respectively) compared to injection-only partnerships. Completing more study interviews was independently associated with lower risk of both RSS and RCU (ARR: 0.92, 95% CI: 0.83–1.00; and ARR 0.91, 95% CI: 0.81–1.00 respectively).

**Table 4:** Multi-level multivariate models for recent high rink injecting behaviors.

	Receptive Syringe Sharing (n = 345)		Receptive Cooker Use (n = 345)	
	RR (95% CI)	ARR (95% CI)	RR (95% CI)	ARR (95% CI)
Injecting only partnerships; no cohabitation†	ref	ref	ref	ref
Injecting only partnership; cohabitated 1–28 days†	1.40 (0.65–2.90)	1.53 (0.5–4.77)	2.69 (1.15–6.28)**	3.37 (1.01–13.42)**
Injecting only partnership; cohabited ≥28 days	4.01 (1.09–14.87)**	4.90 (1.01–24.30)**	2.94 (0.62–13.83)	8.48 (1.55–46.35)**
Sexual injection partnership, cohabitated 1–28 days†	3.62 (1.63–8.04)**	4.70 (1.56–14.05)**	5.26 (1.93–14.34)***	8.62 (2.40–31.30)***
Sexual injection partnerships, cohabitating ≥28 days	3.97 (1.50–10.56)**	5.45 (1.72–17.18)**	2.86 (1.00–8.24)**	7.40 (1.95–28.04)**
Time in study(per interview completed by injection partnership)	0.93 (0.86–1.00)*	0.92 (0.83–1.00)*	0.91 (0.82–1.00)*	0.91 (0.81–1.00)*

Note: Data from 345 HCV-negative observations representing 70 unique partnerships followed monthly for 6-months or until inactive.

GEE models with link logit, controlling for multiple partnerships.

ARR = Adjusted Risk Ratio.

† Prior month.

*p≤0.10.

**p≤0.05.

***p≤0.001.

## Discussion

Our study of HCV-serodiscordant injection dyads is important for the understanding of contextual factors influencing transmission dynamics of viral infections. Contrary to other studies, gender and age composition of the injection partnership and person-level characteristics did not influence injecting behaviors. Instead, injection partnerships also in sexual relationships and cohabitating were at greater risk for both receptive syringe sharing (RSS) and receptive cooker use (RCU); findings that highlight the important role relationship-factors have on individual injecting behaviors. Our study expands on previous findings showing that long-standing and complex relationships, such as those based on economic, social, and sexual ties, are associated with an increase in an individual’s risk of sharing syringes and injecting equipment [37], [38], and demonstrates that these findings extend to HCV-serodiscordant injection partnerships and young PWID. Recognizing the amplified effect of injecting together, cohabitating, and engaging in a sexual relationship has on RSS and RCU identifies the indirect role relationship forces have on individual injection behaviors. Tailoring prevention strategies to not only recognize the role of the partnership on individual risk, but to develop harm reduction strategies focused on close or intimate injection partnerships might reduce transmission of HCV.

The greater risk of RSS and RCU observed in our sample of intimate injection partnerships, those who lived together and were also in a sexual relationship, could be due to an added resource dependence or connectedness. The largest proportion of individuals within this type of injection partnership also reported always pooling money together to purchase drugs and being injected by their “HCV-positive” injection partner (data not shown). Individuals who are dependent on their sexual partner for drugs or injecting practices, can experience limited control over their own injecting behavior [39], contributing to differential power between injection partners. Recently, tools have been developed to measure the influence of relationship power on sexual HIV transmission within sexual partnerships [40], [41]. However, little research has focused on identifying power dynamics within injection partnerships. Traditional gender roles can reinforce power imbalances within injection partnerships [42], [43], potentially encouraging one partner to play a passive role in the shared injection experience. We are unable to assess if relationship power is the mechanism by which disparities in sharing or risk behaviors occur within our sample. However, our data do demonstrate that risk profiles differ depending on the type of injection relationship one is in. Future qualitative studies may better capture the mechanism driving sharing behaviors within intimate injection partnerships.

The HIV field has recognized the critical role that couples play in the sexual transmission of HIV and used this understanding to adapt prevention efforts to also target social-level influences on individual behavior. Couples-based voluntary HIV counseling and testing, in which the couples receive their HIV test results and counseling together, has been shown to be an effective strategy for reducing HIV transmission by initiating behavior change among HIV-discordant sexual partnerships, independent of sexuality [44], [45]. A similar strategy of partner testing and disclosure may prove effective in reducing HCV transmission through changing injection practices among intimate injection partnerships. Additional research examining acceptability of injection partner based HCV-testing and counseling will help inform such efforts.

However, the risk for both RSS and RCU was greatest for injection partnerships in close relations among our sample of HCV-serodiscordant dyads suggests that awareness of an injection partner’s HCV infection status may have limited impact on sharing within such intimate relationships. Interdependence Theory [46] acknowledges that how people exchange rewards and costs in a relationship is influenced by their level of relationship depth. Intimate injection partnerships may be willing to engage in RSS and RCU with the “reward” of increased closeness even at the potential “cost” of HCV transmission, and may explain why findings from the few studies examining the influence of knowing (or perceiving) one’s injection partner’s HCV status on risk behaviors have been mixed [47]–[51]. Even when HCV status is known within injection partnerships, sharing syringes and injecting equipment may be viewed as an extension of trust in the injecting relationships [52], [53] and to increase closeness when injecting with their sexual partner [54], [55]. In a qualitative study of PWID injecting norms in Hungary, Gyarmathy et al. (2006) found that risky injecting behaviors existed even when infection status was disclosed; therefore suggesting that desire for relationship closeness may override infection concerns [56], especially within sexual injection partnerships. While sexual relationships are often characterized by intimacy and closeness, cohabitating and sexual relationships may have been formed on a desire/need for financial or physical safety, or a way to secure drugs.

Our findings should be considered with the following limitations in mind. First, our measures of cohabitation and recent sexual behaviors represent features of relationship closeness or relationship intimacy rather than a direct measure of relationship closeness/intimacy. Currently no validated measure exists to measure intimacy within drug using populations; therefore researchers rely on proxy variables to examine relationship-level factors. Additional work is warranted to develop a validated measurement method for relationship domains to gain further insight into the context of injecting risk. Work that could benefit from applying scales, such as the Dyadic Adjustment Scale used to assess the quality of relationships [57], to study interpersonal risk for HCV transmission. Second, the data were collected by self-report and vulnerable to social desirability bias resulting in under-reporting of risk behavior. However, this effect, if present, would bias measures of association toward the null. Third, while cohabitating and being in a sexual relationship was associated with greater risk of RSS and RCU, and completing more study visits was associated with lower risk, other relationship-level factors that might be perceived as signs of intimacy such as frequency of injecting together, and relationship duration were not found to be predictive. This may reflect statistical power issues due to the limited sample size. Fourth, by requiring that partners injected together at least 5 times in the prior month, our sample was comprised of more stable, established injection partnerships rather than casual, one-time injection partnerships. This may limit the generalizability of our results to less stable injection partnerships. Lastly, information on situational and environmental factors, such as access to harm reduction services, was not available for our analysis. Moreover, a validated measure for relationship intimacy or closeness may help to better understand the injecting relationships studied.

This study suggests a need for: 1) individuals to develop skills to recognize risk and strategies to change behaviors when injecting with someone with whom they are in a relationship; 2) prevention messages that stress the importance of safe injecting practices, including always using a clean cooker, with intimate injection partners rather than just those who are new or untrustworthy; and 3) encouraging couples-based HCV testing and counseling. Individuals engaged in intimate relationships may have a concern for the well-being of the other injection partner, a concern not present in more casual injection partnerships [58]. This unique aspect of the relationship can be harnessed for the purpose of harm reduction.

In conclusion, risk for RSS and RCU differed depending on the type of partnership in which a PWID is engaged. Our data suggest that it is not merely the act of injecting in the same physical location that confers risk. Rather, injecting risk is influenced by the social context and appears to increase when additional relationship layers are introduced. Qualitative or mixed method research may improve our understanding of the underlying mechanisms at play within such multiplex relationships. In the era of test, treat, and retain, PWID in more stable injection partnerships may offer an opportunity to intervene with lasting change.
